# Is HERA a panacea for making the European Health Union a reality?

**DOI:** 10.3325/cmj.2021.62.539

**Published:** 2021-12

**Authors:** Stjepan Orešković

The health information systems in the European Union (EU) are an example of politically ambitious, technically impressive, and driven-by-science mechanisms. Several international indicators and data sets are currently in function, such as Eurostat, World Health Organization Health for All database, and Organisation for Economic Co-operation and Development health data, all serving as broad data platforms. Others have a more specific focus, such as data sets and platforms of the European Monitoring Centre for Drugs and Drug Addiction and European Centre for Disease Prevention and Control (ECDC). More than 60 indicators are already up and running, and data can be disaggregated by sex, age, socio-economic status, and region. The *European Core Health Indicators* (ECHI) data tool offers data on ECHI indicators and other European health indicators, which are available in different formats (1). The whole system and the individual tools seem to be highly efficient, functional, and integrated. However, when on January 30, 2020, the WHO declared a public health emergency of international concern, and a pandemic on March 11, 2020, all data flows and information systems in public health underwent a radical stress test. The wake-up call was very loud, ringing the bell for information bureaucrats working in a giant information balloon. An unprecedented information chaos ensued: the new coronavirus proved to be a challenge completely different from the recent SARS-CoV-1 (2002-2003), Middle East Respiratory Syndrome (2012), and Ebola (2014) epidemics and pandemics. The information structures that were once considered stable and functional were exposed by SARS-CoV-2 as unreliable, fragile, and questionable. Predictions of scientists and institutions, such as the Centers for Disease Control and Prevention (CDC, Atlanta, USA), ECDC (Stockholm, Sweden), or the Institute for Health Metrics (Washington, USA) about the pandemic were sometimes contributing to the growing chaos. Poor predicting models were causing great harm by sometimes leading to devastating decisions about resource allocation and serial lockdowns. As Ioannidis et al put it: “We need models which incorporate multicriteria objective functions. Isolating infectious impact, from all other health, economic, and social impacts is dangerously narrow-minded. More importantly, with epidemics becoming easier to detect, opportunities for declaring global emergencies will escalate. Erroneous models can become powerful, recurrent disruptors of life on this planet. Civilization is threatened by epidemic incidentalomas.” (2). The official sources of information, such as national health information institutes and statistical offices, lost their authority. The loss of authority was also a consequence of the tendency among many working in science (and medicine in particular) to generate “fast food science” or to present science as if it were religion. This applies also to public health policy as an inherently political exercise, as well as to every new health program, which is subjected to ideological biases, political priorities, social environment, and budgetary constraints. For the general public and media, it is now hard to accept that the integrated system of science, public health policy, and health programs is very much a work in progress (3).

In an attempt to manage the information crisis, which turned into an economic and social crisis, the European Commission was looking for ways to get out of the information balloon and tackle the ongoing and forthcoming problems. Ursula von der Leyen, president of the EU Commission, announced a new legislative proposal to create a European health data space. In a mission letter to Health Commissioner Stella Kyriakides, she writes: “I want you to work on the creation of a European health data space to promote health-data exchange and support research on new preventive strategies, as well as on treatments, medicines, medical devices and outcomes. As part of this, you should ensure citizens have control over their own personal data” (4).

To make the European Health Union a reality, the institutional capacity and ability of the European central “government” needs to be strengthened. Thus far, it has been just another EU phrase. However, member states and European Commission are advocating a new approach coming from the position that “no country can effectively prevent or tackle a cross-border public health crisis on its own.” (5). The chosen name for the new “omnipotent” institution is HERA (Health Emergency Preparedness and Response Authority). HERA represents a fundamental shift in the approach of the European Commission to health systems management, an area in which the European Commission was previously reluctant to get involved. The name of the Greek goddess Hera (known by the Romans as Juno) was not chosen by chance. The current health crisis demanded more than summoning the powers of Apollo, Asclepius, Artemis, or Hygiea – the standard “god pack” called for actions in normal times. Exceptional times need a special and powerful remedy. In Greek mythology, Hera, the goddess of women, marriage, and childbirth, used to hold a special power. With Zeus, she reigned on Mount Olympus, and Homer dubbed her “ox-eyed” because of her large, full eyes. Today, HERA will need the same capacities for surveillance, monitoring, and intervention if we want to change how the EU manages the complexity of the institutional infrastructure and inter-governmental relations in Europe. If we add to this the existing behaviorally and socially determined health problems, “Looking ahead to the next major threat to health HERA will be expected to undertake both short-term and long-term horizon scanning to guide policy.” (6). To fully understand the impeding threats to health, HERA will be required to take a One-Health approach. With a budget of over EUR 5.3 billion between 2022 and 2027, this institution may play an important role by “issuing policy guidance to member states, providing an overarching coordination mechanism for the activities of pre-existing EC agencies and institutions related to health emergency preparedness planning and response, anticipating cross-border threats to health and developing countermeasures.” (6). HERA is also expected to “advance science and develop innovative health solutions in specific areas including through applied sciences and digitalization in the area of diagnostics, therapeutics and vaccines. It could also trigger innovation in healthcare systems, increasing their efficiency and supporting novel as well as disruptive technologies within the field of crisis-relevant countermeasures” (7).

The issue of the *Croatian Medical Journal* that you are holding in your real or virtual “hands” is a valuable attempt to generate new data via research focusing on important topics of Europewide interest: treatment of rare diseases, pathways for treatment of HIV, new drug related groups-based mechanisms for financing of acute hospital care, and social media habits and attitudes on e-professionalism of medical and stomatology students (8-11). The presented results may contribute to the content and quality of the “European health data space.” Modern digital platforms for evidence-based policies at the national and EU level require the establishing of a sound connection between data collection, analysis, distribution, and scientific research/interpretation. The articles published in this issue aim to both assess the EU's impact on the Croatian health policy and inform European mechanisms and processes to shape particular health policy at the EU level. Ivčić and Radin (8) focused on the rare diseases policy to obtain an insight into the process of policy shaping, starting at the EU level and moving down to the Croatian national level. Although one might erroneously conclude that “rare” diseases affect only a few people, indicating a low-number low-importance matrix, the opposite is true. These diseases are present in about 6%-8% of the population (27 and 36 million people in the EU), with 5000 to 8000 different diagnoses. The study shows that the implementation of the EU health policy in the area of rare diseases policy involves complex legal, financial, and medical mechanisms. Kalanj et al (9), by directly dealing with the health services efficiency, assessed the impact of the Croatian hospital services funding reform on inpatient care and acute hospitals. The study analyzed resourcing, performance, and financing data for 33 acute hospitals between 2009 and 2018. It assessed hospital activity and diagnosis-related grouping average length of stay; hospital staffing; Croatian Health Insurance Fund revenue streams; and hospital incomes and expenditures. The authors concluded that during the study period the cost efficiency of Croatian public hospitals did not meaningfully improve. The non-attainment may be explained by the failure of reformers to heed the experience of other countries, including a growing number of Central and South-East European countries following the introduction of Australian Refined Diagnosis Related Groups. The experience of these countries showed that a hospital payment reform of this nature calls for systematic and coordinated actions, inter-agency collaboration, and a strategic approach where the various interventions are in congruence and act to reinforce one another. One more example of how improved health information systems can integrate health-services and make them more cost-effective is shown by Beck et al (10) in a study performed at the University Hospital for Infectious Diseases, Zagreb. The authors estimated the cost-effectiveness of the EmERGE Pathway of Care for medically stable people living with HIV (10). The studied pathway includes mHealth application that enables individuals to communicate with their caregivers – the application also monitors the use, cost, outcomes, and impact of treatments. Viskić et al (11) compared social media use and attitudes toward e-professionalism between medical and dental students, and assessed their opinion on potentially unprofessional behavior and posts. The two groups of students mostly differed in their opinion on whether posting patient photos on social media represented unprofessional behavior (61% dental vs 89.8% medical) – another confirmation of the contemporary importance of images. The responsibility of a physician not to reveal any personal information (23% dental vs 41.8% medical) proved again, in the new and challenging digital and social media environment, to be an important component of the traditional physician-patient relationship. Students' high e-professionalism was evidenced by their high sensitivity to the inappropriateness of posting online critical comments about faculty (53% dental vs 39.7% medical). And what about their teachers?
